# Amphiphilic p-Sulfonatocalix[4]arene as “Drug Chaperone” for Escorting Anticancer Drugs

**DOI:** 10.1038/srep09019

**Published:** 2015-03-12

**Authors:** Yi-Xuan Wang, Dong-Sheng Guo, Yong-Chao Duan, Yong-Jian Wang, Yu Liu

**Affiliations:** 1 Department of Chemistry, State Key Laboratory of Elemento-Organic Chemistry, Collaborative Innovation Center of Chemical Science and Engineering (Tianjin), Nankai University, Tianjin 300071, P. R. China; 2 College of Life Sciences, Collaborative Innovation Center of Chemical Science and Engineering (Tianjin), Nankai University, Tianjin 300071, P. R. China

## Abstract

Supramolecularly constructing multifunctional platform for drug delivery is a challenging task. In this work, we propose a novel supramolecular strategy “drug chaperone”, in which macrocyclic amphiphiles directly coassemble with cationic drugs into a multifunctional platform and its surface is further decorated with targeting ligands through host–guest recognition. The coassembling and hierarchical decoration processes were monitored by optical transmittance measurements, and the size and morphology of amphiphilic coassemblies were identified by dynamic light scattering and high-resolution transmission electron microscopy. In cell experiments to validate the drug chaperone strategy, the anticancer activities of free drugs were pronouncedly improved by coassembling with amphiphilic chaperone and further functionalization with targeting ligand.

In the past decades, nanotechnology has played important roles in many fields, including materials science, photonics and drug delivery. In medical area, nanotechnology focuses on the development of methodologies for delivering medicinally active molecules to the site of disease with maximized therapeutic benefits but minimized systemic toxicity and undesired side effects for enhanced patient compliance^1^,^2^. A great deal of effort has been devoted to the fabrication of nanovehicles that serve as efficient diagnostic and/or therapeutic platforms for tumor-targeted drug delivery, which include liposomes, polymer nanoparticles, dendrimers and some inorganic materials^3^,^4^. An ideal nanovehicle may feature: (1) biocompatibility/biodegradability for safe administration; (2) feasible synthesis with high yield and purity; (3) facile encapsulation of multiple diverse drugs with high loading efficiencies to reduce the systemic toxicity and extra burden for the patients to excrete the carriers; (4) facile functionalization, in particular for the display of suitable targeting ligands on the surface to achieve the “active targeting” to specific cells or tissues^5^,^6^. Most of the nanovehicles reported so far employ one of the two loading approaches, i.e. non-covalent^7^ and covalent^8^,^9^,^10^. In the former approach, drug molecules are loaded within nanovehicles via physical encapsulation, which could alter the drug's pharmacokinetic properties and biodistribution profiles but show generally low loading efficiencies of less than 10%. In the latter approach, drug molecules, generally serving as hydrophobic blocks, are conjugated to hydrophilic synthetic blocks (usually hydrophilic polymer) and the resulting drug amphiphiles self-assemble into drug-loaded nanoparticles. This approach often requires tedious syntheses of modest yields, as well as further functionalization by imaging probes and targeting ligands. In both approaches, complicated multistep synthesis has to be repeated when altering any of the components incorporated^11^. Therefore, it is still a serious challenge to develop an efficient strategy for constructing multifunctional targeted platform that is capable of binding different drugs with high loading efficiencies yet readily functionalizable with various tags for targeting cancer cells. Nicolas *et al.* recently constructed a multifunctional polymeric platform, which simultaneously processes targeting ligands for cancer cells and specific antibodies for the biomarker of Alzheimer's disease^12^. The resulting nanoparticles were successfully used to target both of the two pathologies. More recently, Yan et al. reported a drug self-delivery system, in which amphiphilic drug–drug conjugate self-assembles into nanoparticles and the resulting nanoparticles are delivered by themselves without necessitating any carriers^13^.

Macrocyclic amphiphiles, composed of a hydrophilic macrocyclic framework and multiple hydrophobic tails, are attracting increasing attention as novel amphiphilic tectons. The macrocyclic amphiphiles exhibit much better performances than the monomeric counterparts^14^,^15^,^16^ and more importantly keep the inherent hydrophobic cavity unoccupied for further guest-binding, thus being commended as amphiphiles with recognition site^17^,^18^. Taking the preorganized framework and the binding ability into account, we envisage macrocyclic amphiphile as a new key component for constructing desired amphiphilic assemblies for diverse applications.

Amongst various macrocycles available, calixarene is particularly attractive, since it can be readily derivatized to macrocyclic amphiphiles by introducing hydrophilic groups at one rim and hydrophobic groups at the other rim, and the intrinsic truncated-cone shape and the rigidity of calixarene framework are beneficial to the stability of amphiphilic aggregation^14^,^19^. Although a variety of calixarene-based amphiphiles have already been employed in many studies, most of them have focused on the self-assembling behavior^20^. In contrast, the coassembling with other amiphiphiles is less explored. Raston *et al.* investigated the coassemblig behavior of anionic and cationic calixarene amphiphiles^21^. When mixed together, these two oppositely charged calixarene amphiphiles coassemble into large vesicles, despite that each of the calixarenes self-assembles into much smaller micelles under comparable conditions. In the present study, we propose a novel targeted drug delivery system “drug chaperone” on the basis of this coaasembling strategy, in which the amphiphilic *p*-sulfonatocalixarene plays dual roles of fabricating self-containing nanovehicle by coassembling with cationic drug and of anchoring a targeting ligand in its cavity for escorted and targeted delivery of the drug. Mitoxantrone·HCl and irinotecan·HCl (Fig. 1) were chosen as cationic drugs, both of which have shown significant clinical effectiveness in the treatment of cancers^22^,^23^. Once coassembled with the amphiphilic drug chaperone, these small molecule drugs are expected to be protected from the premature degradation^24^ and then the nanosized vehicles target cancerous tissues as a consequence of the passive accumulation by the tumors' enhanced permeability and retention (EPR) effect^25^. However, the binding capability of amphiphilic calixarene assemblies has not intentionally been utilized in the foregoing studies. In the present study, we propose to decorate the amphiphilic coassembly surface with targeting ligands via host–guest recognition to achieve the “active targeting” to specific cells or tissues. Since the decoration is performed in a noncovalent manner, the ligand can be readily altered or potentially combined with other ligand while the coassembly remains unaffected. By coassembling cationic drug with amphiphilic calixarene into nanovehicle and further anchoring targeting ligand on its surface, the anticancer drug is safely escorted and efficiently delivered to the targeted cancer cells.

## Results and Discussion

**Figure 1:**
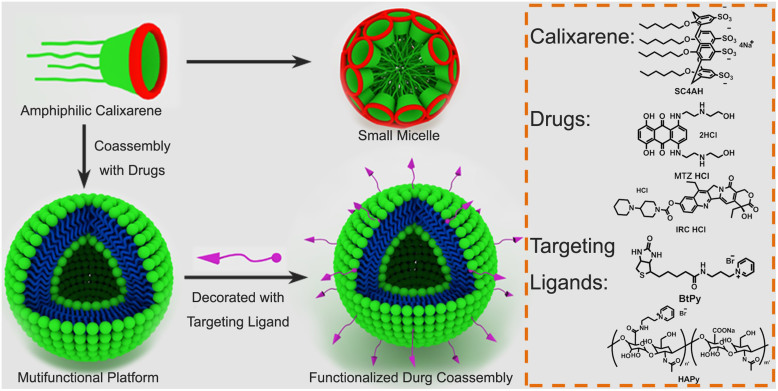
Functionalization protocol of the “drug chaperone” strategy and chemical structures of anticancer drugs (IRC and MTZ), SC4AH and targeting ligands (HAPy and BtPy).

The coassembly of anionic and cationic (catanionic) amphiphiles offers an efficient approach for constructing complicated self-assembled nanostructures^26^. Owing to the strong electrostatic attraction between the oppositely charged head-groups, the catanionic amphiphiles pack more densely in the coassembly with a reduced head-group area, leading to spontaneous formation of stable and large assemblies. In addition to the catanionic calixarene amphiphile constructed by Raston *et al.*^21^, amphiphilic calixarenes can also be embedded in liposomes for protein sensing^27^. We envisage that amphiphilic calixarenes coassemble with drug molecules to play an essential part of the “drug chaperone” strategy, which features: (1) high loading efficiencies achieved with minimized amounts of non-drug components; (2) precise control of the drug content to minimize the batch-to-batch variation, which is enabled by the self-adaptive property of amphiphilic assembly; (3) protection of drugs from premature degradation and delivery to cancerous tissues by passive accumulation via EPR effect; and most importantly, (4) a versatile and multifunctional nano-platform that can be further functionalized with various targeting ligands in a facile, non-destructive, modular and noncovalent manner via host–guest chemistry to achieve the “active targeting” to specific cells or tissues (Fig. 1).

In this study, we first fabricated such coassemblies by using amphiphilic calixarenes and two cationic drugs. Irinotecan·HCl (IRC) is an amphiphilic prodrug that totally aggregates at concentrations higher than 2 mM^28^. Mitoxantrone·HCl (MTZ) is more hydrophilic due to the two positively charged nitrogen atoms and is expected to show stronger electrostatic interactions with negatively charged calixarenes. Since the hydrophilic macrocyle of *p*-sulfonatocalix[4]arene tetrahexyl ether (SC4AH) is predisposed to locate on the surface of coassembly, we then fuctionalized the coassembly by introducing crosslinkers or targeting ligands onto the surface. Finally, the anticancer activities of calixarene–drug coassemblies and their potential utility in targeting therapeutic application were assessed in vitro.

### Fabrication of Calixarene–Drug Coassembly

**Figure 2:**
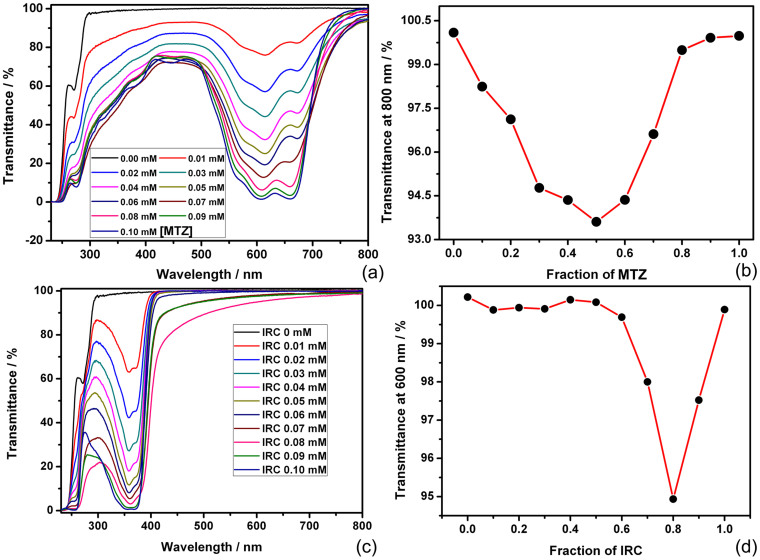
(a) Optical transmittance of SC4AH–MTZ mixtures of varying fractions, while [MTZ] + [SC4AH] = 0.1 mM. (b) Dependence of the optical transmittance at 800 nm on the fraction of MTZ. (c) Optical transmittance of SC4AH–IRC mixtures of varying fractions, [IRC] + [SC4AH] = 0.1 mM. (d) Dependence of the optical transmittance at 600 nm on the fraction of IRC.

Carrying hydrophilic sulfonates at the upper rim of calixarene and the hydrophobic n-hexyl chains at the lower rim as well as the intrinsic cone-shaped head-group suitable for high-curvature aggregation, SC4AH is expected to exhibit the desired amphiphilic behavior. Indeed, SC4AH forms a micellar assembly with a critical micelle concentration of ca. 0.5 mM^19^. In order to determine the coassembling stoichiometry and also to find the optimum mixing ratio, the Job analysis was performed for the assembly-induced decrease of optical transmittance at 800 nm obtained for a series of solutions at a total concentration ([SC4AH] + [drug]) of 0.1 mM, where neither SC4AH nor drug molecule could form any nanostructure by itself. As shown in Fig. 2a–b, the transmittance at 800 nm exhibited a fairly broad shape, indicating formation of coassembly in a rather wide range of the MTZ molar fraction over 0.1–0.7 with a peak at 0.5. To balance the efficiencies of coassembling and drug loading, the MTZ molar fraction of 0.6 ([SC4AH] = 0.04 mM, [MTZ] = 0.06 mM) was chosen throughout the work, unless mentioned otherwise. Since MTZ is a dication and SC4AH is a tetraanion, multivalent electrostatic attraction should operate upon coassembling to reduce the effective charge on MTZ, allowing its stacking as indicated by hypochromic effect (Fig. S4a)^29^. In addition, the absorption bands of MTZ at 610 and 660 nm were bathochromically shifted by 6–10 nm to higher wavelengths, demonstrating that SC4AH–MTZ complexes formed supra-amphiphilic coassembly and MTZ molecules were located in hydrophobic medium with smaller polarity than the bulk water environment^30^.

For the SC4AH–IRC system, the Job plot of the transmittance at 600 nm showed a sharp peak at a high IRC molar fraction of 0.8, or a SC4AH/IRC molar ratio of 1:4 (Fig. 2c–d) in nice agreement with the charge ratio. The contrasting behavior in Job plot of IRC from that of MTZ may be attributed to the weaker electrostatic interaction of monocationic IRC with tetraanionic SC4AH, compared to dicationic MTZ, which allows their coassembling only near the exact molar ratio at which the charges are balanced.

Unlike the *p*-sulfonatocalix[4]arene (SC4A)–IRC system reported previously^31^, IRC hardly penetrates into the calixarene cavity of SC4AH, because the alkylation of SC4A at its lower rim makes the SC4AH framework more rigid and pinched^32^. The coassembling behavior of SC4AH with IRC was examined by UV/Vis spectroscopy to show a significant hypochromic effect on the IRC absorption upon addition of SC4AH (Fig. S5a), due to the alignment of the aromatic chromophores as was the case with the self-association^28^. This behavior is in sharp contrast to the remarkable hyperchromic effect observed upon addition of SC4A to a solution of IRC, which is caused by the penetration of IRC into the SC4A cavity that prevents coassembling^31^. Furthermore, no cross peaks were observed between the IRC and SC4AH protons in the ROESY spectrum (Fig. S5b). All of these results demonstrate that IRC does not penetrate into the calixarene cavity but forms an ion-pair complex with SC4AH, which in turn self-aggregate to give an SC4AH-IRC coassembly. Crucially, the absorption maximum of IRC was bathochromically shifted upon addition of SC4AH, indicating more hydrophobic environment around the chromophore, and the CD intensity of IRC was enhanced (Fig. S6), suggesting closer packing and conformational fixation in the coassembly^28^, Considering all the results, we conclude that SC4AH and IRC form amphiphilic coassembly when mixed together in 1:4 ratio and hence the concentrations of 0.01 mM SC4AH and 0.04 mM IRC, respectively, were employed throughout the work, unless noted otherwise.

Control experiments showed no decrease of the transmittance at 800 for free SC4AH, MTZ, IRC, and the host fragment 4-(heptyloxy)benzenesulfonate (SHS) under the comparable conditions (Fig. S4), indicating that none of these drugs, host, or host fragment can form large aggregates and the cyclic tetramer structure of SC4AH is crucial to induce the amphiphilic aggregation.

Since the electrostatic interaction is the major driving force for complexation and coassembling, the effects of solution pH on the stability of coassembly was assessed. Somewhat unexpectedly, the SC4AH–IRC and in particular the SC4AH–MTZ particles were totally stable even in alkaline solutions (Fig. S7), eventually exhibiting excellent stabilities over a wide range of pH from 2 to 10. This is probably because the acidities of protonated MTZ and IRC are significantly reduced and not deprotonated even at pH 10 in the hydrophobic environment of coassembly. Thus, despite the dynamic equilibrium between the coassembled and unassembled states of the positively charged drug molucules^33^, only the free species can be deprotonated at alkaline pHs. The complexation driven by multivalent charge interactions favors ionic states, thereby preventing coassembled species from deprotonation^34^. In other words, the protonated drugs were protected from the alkaline conditions by coassembling with amphiphilic chaperones.

**Figure 3:**
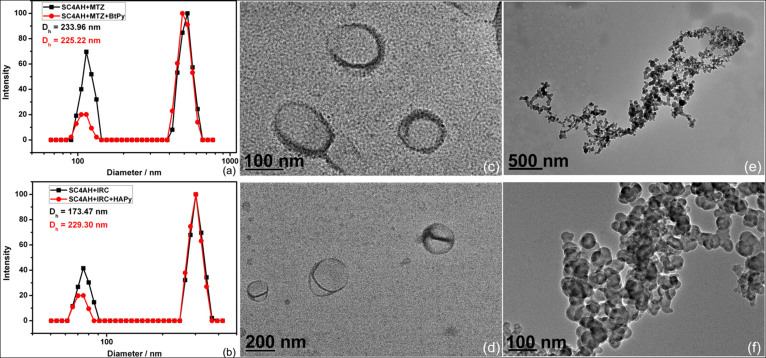
Size distributions determined by scattered light intensity of (a) SC4AH–MTZ coassembly with and without BtPy (0.04 mM) and (b) SC4AH–IRC coassembly with and without HAPy (0.01 mM) measured by DLS. TEM images of (c, d) SC4AH–MTZ coassembly and (e, f) SC4AH–IRC coassembly.

Dynamic light scattering (DLS) and high-resolution transmission electron microscopy (TEM) were performed to identify the size and morphology of the amphiphilic coassembly. DLS measurements at a scattering angle of 90° revealed that the average hydrodynamic diameter is 234 nm for the particles derived from SC4AH and MTZ (Fig. 3a) but 173 nm for those from SC4AH and IRC (Fig. 3b). According to the size distribution determined by the number of particles, the majority of SC4AH–MTZ particles are ca. 100 nm in diameter, while SC4AH–IRC particles are ca. 70 nm (Fig. S8).

The TEM images of SC4AH–MTZ coassembly showed the hollow spherical morphology with a diameter ranging from 100 to 200 nm (Fig. 3c–d), which is consistent with the DLS result. From the distinguishably dark periphery and the light central parts, we obtained the thickness of the membrane as ca. 6 nm, which is in the same order of magnitude as a sum of four SC4AH or MTZ lengths, suggesting formation of binary lamellar structure. In contrast, the TEM images of SC4AH–IRC coassembly showed solid spherical morphology with a diameter ranging from 50 to 100 nm (Fig. 3e–f), which is also in agreement with the DLS result. Furthermore, both SC4AH–MTZ and SC4AH–IRC nanoparticles were found to possess negative zeta potentials (Fig. S9), indicating that sulfonate groups of SC4AH are on the surfaces of the nanoparticles. In other words, the calixarene cavities are positioned on the surfaces for further decoration with various tags via host−guest interactions with SC4AHs.

**Figure 4:**
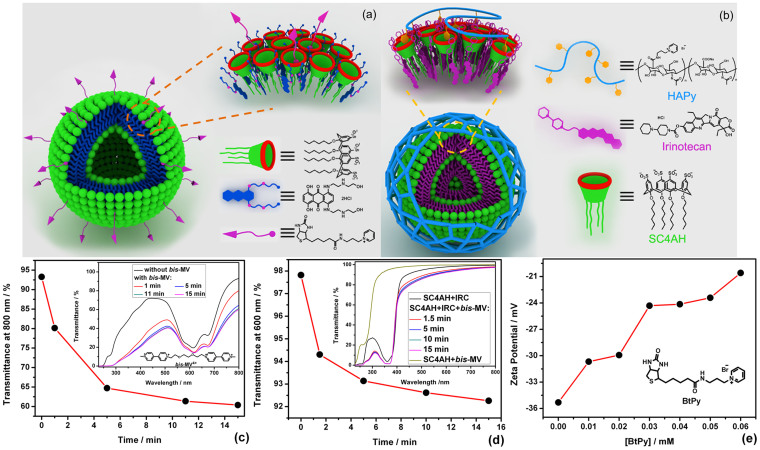
Schematic illustrations of (a) SC4AH–MTZ coassembly and (b) SC4AH–IRC coassembly. (c) Dependence of the optical transmittance of SC4AH–MTZ coassembly at 800 nm on time in the presence of bis-MV (0.04 mM). (d) Dependence of the optical transmittance of SC4AH–IRC coassembly at 600 nm on time in the presence of bis-MV (0.02 mM). [IRC] = 0.08 mM, [SC4AH] = 0.02 mM. (e) Dependence of the zeta potential of SC4AH–MTZ coassembly on BtPy concentration.

Combining all of the aforementioned results, we deduced that the coassembled nanoparticles exhibit typical amphiphilic characteristics. SC4AH and drug molecule are put together by electrostatic and hydrophobic interactions. The intrinsic electrostatic repulsion between the positively charged drug molecules is replaced by the electrostatic attraction between the oppositely charged drug and SC4AH molecules upon coassembling. The hydrophobic alkyl chains in SC4AH are packed together to prove a hydrophobic environment suitable for the π–stacking interaction of the aromatic moiety of drug molecule. Schematic illustrations of the coassembly models for SC4AH−MTZ and −IRC are shown in Fig. 4a–b. Crucially, the particles were isolated by ultracentrifugation and subjected to ^1^H NMR spectral analyses^16^. The amphiphile:drug molar ratio of the precipitates obtained by ultracentrifugation was 1:1.82 (loading efficiency: 43.0%) for SC4AH–MTZ coassembly and 1:3.43 (loading efficiency: 65.2%) for SC4AH–IRC coassembly. The chemical composition of the nanoparticles close to the original mixing ratio could be attributed to the effective binary coassembling.

### Surface functionalization

Having constructed the calixarene–drug coassemblies, we further investigated the host−guest recognition behavior of the calixarenes located on the particle surface. Several pyridinium and viologen guests were selected as functional tags, bis-MV as a crosslinker of particles, and BtPy and HAPy as targeting ligands for cancer therapy. Since MV can form stable stoichiometric 1:1 complexes with amphiphilic *p*-sulfonatocalixarene^35^, bis-MV is expected to crosslink nanoparticles via host−guest interactions. The crosslinking behavior was monitored by optical transmittance measurements (Fig. 4c–d). In the presence of bis-MV, the optical transmittance of coassemblies decreased significantly. In a control experiment, the addition of bis-MV to a solution of SC4AH did not cause appreciable turbidity, indicating no formation of large aggregates. These experiments clearly proved that the hierarchical aggregation occurred due to the noncovalent crosslinking of coassemblies by bis-MV. It should be noted that, benefiting from the abundant host−guest binding sites on the surface and the strong host−guest interactions between MVs and calixarenes, the rate and extent of crosslinking are both remarkable, indicating that the noncovalent decoration of the surface is fast and efficient.

To realize the functionalization of coassemblies, biotin–pyridinium (BtPy, for SC4AH–MTZ coassembly) and hyaluronic acid–pyridinium (HAPy, for SC4AH–IRC coassembly) were synthesized as targeting ligands. Pyridinium was chosen as a binding moiety for amphiphilic *p*-sulfonatocalixarene instead of viologen because the high toxicity of viologen poses considerable risks to human health^36^. ^1^H NMR measurements were performed to explore the binding behavior of pyridinium tags with calixarene. As shown in Fig. S10, the proton signals of pyridinium exhibited pronounced upfield shifts due to the ring current effect of the aromatic nuclei of calixarene^37^, whilst those of biotin, hyaluronic acid or IRC were not affected appreciably. These results demonstrate that the pyridinium moieties are encapsulated into the calixarene cavity while the functional moieties stay outside, which ensures that their original functions remain intact.

Zeta potential measurements were performed to identify the introduction of targeting ligands onto the surface of coassemblies. As shown in Fig. 4e, the zeta potential value of SC4AH–MTZ coassembly gradually increased from −35 mV to −21 mV upon addition of BtPy, due to the electrostatic compensation between the oppositely charged head-groups. This clearly indicates that BtPy was successfully introduced onto the surface of coassembly via host–guest interactions. In comparison to BtPy, HAPy possesses multiple negative charges. Upon incubation with SC4AH–IRC coassembly, the zeta potential of the coassembly decreased from −20 mV to −30 mV (Fig. S11), which is ascribable to the successful noncovalent decoration. However, continuous addition of HAPy did not lead to further decrease of zeta potential, which is possibly due to the electrostatic repulsion between hyaluronic acid and SC4AH–IRC coassembly. It should also be noted that both coassemblies still keep negative surface charges even after surface functionalization, which is favorable for drug delivery, because negatively charged nanoparticles exhibit less unspecific cell uptake and good protein resistance^38^.

Control experiments revealed that after treatment with targeting ligands the coassemblies did not exhibit any apparent change in turbidity and size distribution (Fig. 3a, 3b, S8 and S12), demonstrating the the non-destructive nature of the surface modification. Combining the aforementioned results, we may conclude that the surface of comassembly is successfully decorated with various ligands in a facile, non-destructive and non-covalent manner by using host–guest chemistry. The ligand can be readily altered while the coassembly remains unaffected, which endows this “drug chaperone” strategy great potential in targeted cancer therapy. In addition, since the species and contents of targeting ligands are readily manipulable, this strategy can be extend to the design of combinatorial platform for anticancer drugs in which the usage of targeting ligands and other functional tags can be sifted and further optimized.

Since irinotecan is a human carboxylesterase 2-active prodrug^39^, its coassembly with amphiphilic *p*-sulfonatocalix[4]arene can be dissipated via the hydrolysis of irinotecan catalyzed by carboxylesterase, which is overexpressed in many tumor cells and tissues^40^. The biodegradation of SC4AH–IRC coassembly in vitro was investigated by performing the enzymatic hydrolysis of IRC. As shown in Fig. S13, the optical transmittance of SC4AH–IRC coassembly increased dramatically while the scattered-light intensity decreased after the treatment with carboxylesterase. Moreover, no spherical structure of coassembly was observed in the TEM image, in nice agreement with the disappearance of the Tyndall effect. These results jointly indicated that the obtained SC4AH–IRC coassembly was dissipated as soon as IRC was specifically hydrolyzed upon exposure to carboxylesterase. The hydrolysis rate of the binary coassembly by CES is much slower than that of free IRC, also because there is a dynamic equilibrium between the assembled and unassembled states of IRC and CES attacks only the free species.

### Cytotoxicity against Cancer Cells

**Figure 5:**
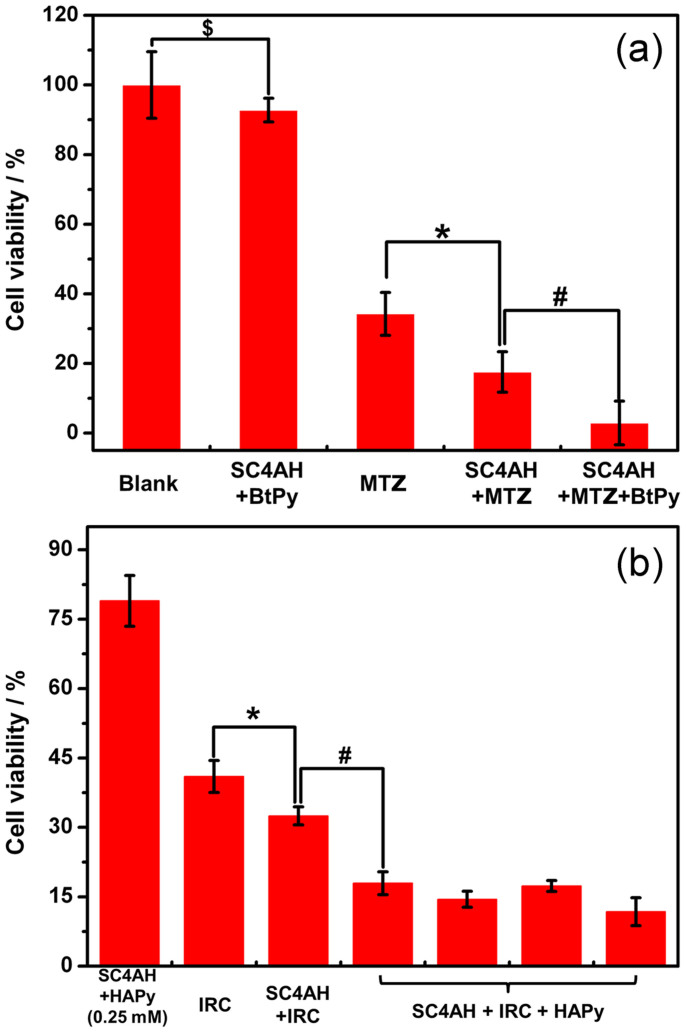
(a) Cell viability of MCF7 cells after exposure to SC4AH–MTZ coassembly for 48 h at 37°C. [MTZ] = 0.01 mM, [SC4AH]:[MTZ]:[BtPy] = 2:3:2. ^$^*P* > 0.05, *P < 0.01 and ^#^*P* < 0.05. (b) Cell viability of MCF7 cells after exposure to SC4AH–IRC coassembly for 48 h at 37°C. [IRC] = 0.05 mM, [SC4AH]:[IRC] = 1:4. *P < 0.01 and ^#^*P* < 0.001. In the SC4AH + IRC + HAPy entry, [HAPy] = 0.0125, 0.05, 0.1 and 0.25 mM, respectively (from left to right).

To evaluate the anticancer activities of calixarene–drug coassemblies and their potential utility in targeting therapeutic application, cytotoxicity experiments were performed in vitro and MCF-7 cell line (a type of human breast cancer cells that abundantly overexpress both biotin^12^ and hyaluronic acid^41^ receptors on the cell surface) was chosen for the assay. For the SC4AH–MTZ system (Fig. 5a), the cell viability of MCF-7 treated by SC4AH–MTZ coassembly is 18%, which is lower than free MTZ (34%). Compared with SC4AH–MTZ nanoparticle which can be internalized into cancer cells via endocytosis, free MTZ is too hydrophilic to penetrate into the lipid bilayer of the cell membrane, resulting in lower anticancer activity. After decorated with BtPy, the SC4AH–MTZ coassembly showed the best anticancer activity while SC4AH with BtPy was practically nontoxic. The biotin moieties on the surface of the coassembly specifically recognize MCF-7 cancer cells by strongly binding to biotin receptors on the cell surface and the coassemblies enter cells through receptor-mediated endocytosis. As a result, the biotinylated SC4AH–MTZ coassembly shows the best anticancer activities toward MCF-7 cancer cell lines. For the SC4AH–IRC system (Fig. 5b), we got similar results that the SC4AH–IRC coassembly decorated with HAPy showed the best anticancer activities due to the efficient internalization via receptor-mediated endocytosis. Upon increasing the concentration of HAPy, the anticancer activities of SC4AH–IRC coassembly remained almost unchanged, indicating that excess HAPy could not bind to the coassembly. It is in nice agreement with our previous deduction from the zeta potential experiments, and also proves that only HAPy exhibit scarcely anticancer activities. Moreover, it is noted that the highly excessive HAPy may disassemble the SC4AH–IRC conjugate to reduce the drug activity, whereas the amphiphilic coassembly could lose the targeting ability with insufficient HAPy. Therefore, the HAPy concentration should be optimized to ensure both the stability and targeting ability of the resulting delivery system.

## Conclusions

In conclusion, we developed a novel strategy of “drug chaperone”, where drug entrapment was successfully achieved by directly coassembling amphiphilic macrocycles with drugs via electrostatic and hydrophobic interactions. The resulting nanoparticles possess high loading efficiencies and protect drug molecules from alkalization. Furthermore, the coassembly serves as a versatile and multifunctional nano-platform that can be hierarchically decorated in a facile, non-destructive and modular manner benefiting from the intrinsic recognition site of macrocyclic amphiphilie. After decorated with targeting ligands, the ternary nanoparticles showed enhanced anticancer activities. It can be envisaged that decoration with other functional moieties will endow the coassembly with new functions, like imaging probes for diagnostics and PEGs for prolonging circulation time in blood. We believe that the present supramolecular strategy of “drug chaperone” based on macrocyclic amphiphilies would open novel avenues to build versatile drug delivery platforms with desired performance and further enables the combinatorial search for the optimum combination of anticancer drug or imaging probe and targeting ligand for diagnosis and therapeutics.

## Methods

All the technical details and procedures are provided in the Supplementary Information.

## Author Contributions

W.Y.X. synthesized the functionalized drug coassemblies. W.Y.X. and G.D.S. performed the functional analyses of the coassemblies. D.Y.C. and W.Y.J. carried out the cytotoxicity studies. All authors discussed the results. W.Y.X. and G.D.S. wrote the main manuscript. W.Y.X. prepared figures 1–5. L.Y. supervised the work and edited the manuscript. All authors reviewed the manuscript.

## Supplementary Material

Supplementary Information SUPPLEMENTARY INFO
