# Supplementary material for: Amphiphilic p-Sulfonatocalix[4]arene as “Drug Chaperone” for Escorting Anticancer Drugs
Source: Sci Rep. 2015 Mar 12;5:9019. doi: 10.1038/srep09019 (PMC4356970; doi:10.1038/srep09019)
Supplement: Supplementary Information — SUPPLEMENTARY INFO [file srep09019-s1.doc]

Supporting Information

**Amphiphilic p-Sulfonatocalix[4]arene as “Drug Chaperone” for Escorting Anticancer Drugs**

Yi-Xuan Wang,† Dong-Sheng Guo,† Yong-Chao Duan,‡ Yong-Jian Wang,‡ and Yu Liu*,†

†Department of Chemistry, State Key Laboratory of Elemento-Organic Chemistry, ‡College of Life Sciences, Collaborative Innovation Center of Chemical Science and Engineering (Tianjin), Nankai University, Tianjin 300071, P. R. China

*E-mail: yuliu@nankai.edu.cn.

Fax: +86-22-23503625.

**General Experimental Section**

**Materials.** Mitoxantrone·HCl (MTZ) and Irinotecan·HCl (IRC) (both from Dalian Meilun Biology Technology), sodium 3-(trimethylsilyl)-1-propanesulfonate (DSS) (from Aldrich), carboxylesterase-2 protein (CES) (from Sino Biological), 3-bromopropylamine·HBr, *N*-(3-dimethylaminopropyl)-*N*′-ethylcarbodiimide·HCl (EDC) and *N*-hydroxysuccinimide (NHS) (both from Aladdin), and sodium hyaluronate (MW = 110000) (from Bloomage Freda Biopharm) were used without further purifications. Sodium 4-(heptyloxy)benzenesulfonate (SHS), *p*-sulfonatocalix[4]arene tetrahexyl ether (SC4AH)[[1]](#endnote-2) and bis-MV[[2]](#endnote-3) were synthesized and purified according to the procedures reported previously.

**Synthesis.**

**Scheme S1.** Synthesis of HAPy[[3]](#endnote-4) and BtPy.

**PYA.** 3-Bromopropylamine hydrobromide (2.0 g, 9.1 mmol) and pyridine (2.0 mL) were dissolved in anhydrous acetone (50 mL), and the mixture was heated to reflux for 8 h under a nitrogen atmosphere. After cooling to room temperature, the precipitates produced were filtered and washed with anhydrous acetone. The product was dried overnight under vacuum (yield: 1.4 g, 51%). ^1^H NMR (400 MHz, D_2_O): *δ* 8.79 (d, *J* = 6.0 Hz, 2H), 8.48 (t, *J* = 7.9 Hz, 1H), 8.00 (t, *J* = 6.8 Hz, 2H), 4.63 (t, *J* = 7.7 Hz, 2H), 3.07–2.99 (m, 2H), 2.43–2.25 (m, 2H).

**BtPy.** EDC (475 mg, 2.48 mmol) and NHS (283 mg, 2.46 mmol) were added to a solution of biotin (500 mg, 2.05 mmol) in DMF (40 mL) and the mixture was stirred at 35 °C for 24 h under a nitrogen atmosphere. PYA (596 mg, 2.00 mmol) and Et_3_N (300 μL) were added to the mixture. The mixture was stirred at 35 °C for another 24 h and evaporated under a reduced pressure to remove DMF. The residue was purified by column chromatography (CHCl_3_:MeOH = 4:1) to yield BtPy (490 mg, 54%) as hygroscopic powders. HR-MS: m/z 363.1852 ([BtPy − Br]^+^, calcd. for C_18_H_27_N_4_O_2_S^+^, 363.1849). ^1^H NMR (400 MHz, D_2_O): *δ* 8.73 (d, *J* = 5.9 Hz, 2H), 8.42 (t, *J* = 7.8 Hz, 1H), 7.94 (t, *J* = 7.0 Hz, 2H), 4.51 (t, *J* = 7.3 Hz, 2H), 4.45 (dd, *J* = 7.8, 4.9 Hz, 1H), 4.27 (dd, *J* = 7.9, 4.5 Hz, 1H), 3.25–3.10 (m, 3H), 2.83 (dd, *J* = 13.1, 4.9 Hz, 1H), 2.60 (d, *J* = 13.1 Hz, 1H), 2.21–2.05 (m, 4H), 1.66–1.36 (m, 4H), 1.35–1.18 (m, 2H). ^13^C NMR (400 MHz, D_2_O): *δ* 177.0, 165.3, 145.9, 144.3, 128.3, 62.1, 60.2, 59.4, 55.4, 39.7, 35.8, 35.4, 30.1, 28.0, 27.7, 25.0.

**HAPy.** EDC (75.6 mg, 0.875 mmol) and NHS (46.7 mg, 0.875 mmol) were added to a solution of sodium hyaluronate (299.6 mg, 2.17μmol) in phosphate buffer solution (PBS, 0.1 M, pH 7.2, 30 mL), and the mixture was stirred at 25 °C for 30 min. Then PYA (77.1 mg, 0.0625−0.5 mmol) was added, and the mixture was stirred for 24 h at room temperature. The resulting solution was dialyzed against an excess amount of deionized water for 5 days. After being freeze-dried, HAPy was obtained as white powder. ^1^H NMR (400 MHz, D_2_O): *δ* 8.80 (d, *J* = 5.1 Hz, 0.12H), 8.48 (t, *J* = 8.6 Hz, 0.05H), 8.00 (t, *J* = 5.8 Hz, 0.11H), 4.63 (t, *J* = 5.5 Hz, 0.27H), 4.42−4.33 (m, 1.84H, H of HA), 3.96−3.22 (m, 10.25H, H of HA), 3.02 (t, *J* = 7.4 Hz, 0.24H), 2.37−2.26 (m, 0.17H), 1.90 (s, 3.00H, H of methyl group of HA). [HAPy] is defined as the concentration of pyridinium moiety which can be calculated by comparing the integrated peak area of the pyridinium protons versus that of the *N*-acetyl protons of HA at 1.90 ppm.

**Measurements.**

**UV/Vis spectroscopy.** The optical transmittance of the aqueous solution was measured at 25 °C in a quartz cell (light path 10 mm) on a Shimadzu UV-3600 spectrophotometer equipped with a PTC-348WI temperature controller.

**NMR spectroscopy.** ^1^H NMR spectra were recorded on a Bruker AV400 spectrometer in D_2_O at 25 °C

**High-resolution TEM measurement.** High-resolution TEM images were acquired using a Tecnai 20 high-resolution transmission electron microscope operating at an accelerating voltage of 200 kV. The sample for TEM measurements was prepared by dropping a sample solution onto a copper grid. The grid was then air-dried.

**DLS measurement.** Solution samples were examined on a laser light scattering spectrometer (BI-200SM) equipped with a digital correlator (TurboCorr) at 636 nm at a scattering angle of 90°. The hydrodynamic diameter (D_h_) was determined by dynamic light scattering experiments at 25 °C.

**Zeta potential measurements.** Zeta potential values were determined at 25 °C on a Brookheaven ZetaPALS (Brookheaven Instrument, USA). The instrument utilizes phase analysis light scattering to provide an average over multiple particles. Doubly distilled water was used as the background electrolyte for zeta potential measurements.

**Cytotoxicity studies.** Human breast adenocarcinoma cells (MCF-7) were cultured in Dulbecco’s modified Eagle’s medium (DMEM) containing 10 vol% FBS at 37 °C in a humidified 5% CO_2_/95% air atmosphere. The cytotoxicity of coassemblies was investigated by MTT [3-(4,5-dimethylthiazol-2-yl)-2,5-diphenyl tetrazolium bromide] viability test on MCF-7. Briefly, cells were seeded in a 96-well plate (2 000 cells/well) with DMEM supplemented with 10% fetal bovine serum and allowed to adhere for 24 h. The cells were then treated with medium-containing coassemblies. Medium without coassemblies was used as a negative control. After 48 h of incubation at 37 °C in 5% CO_2_, 10 μL of MTT solution (5 g⋅L^-1^ in phosphate buffer) were added to each well and the cells were incubated further for 4 h. After the removal of the medium, the cells were washed with phosphate buffer and dissolved in DMSO. The OD was measured at a wavelength of 490 nm with a Multiskan Ascent plate reader. Each sample was replicated in five wells.

**Supplementary Figures**


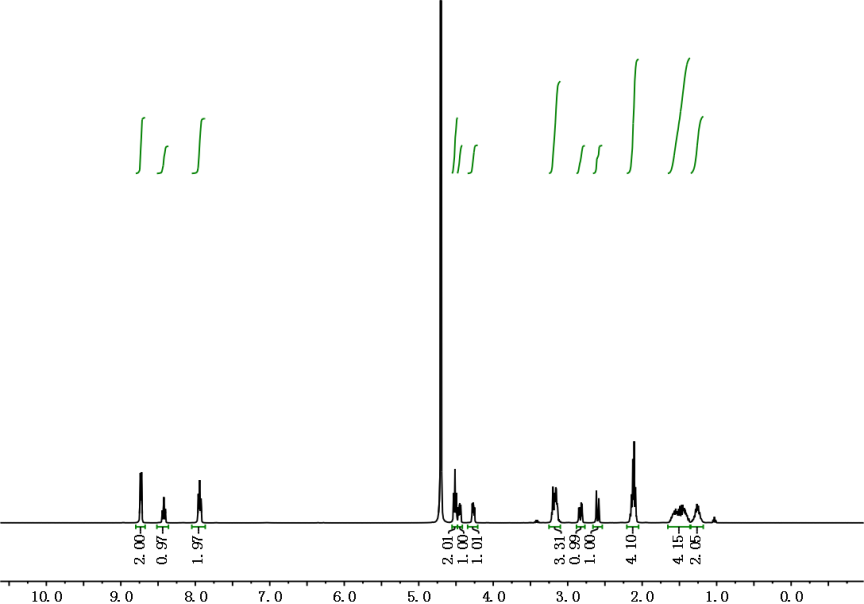


**Figure S1.** ^1^H NMR spectrum (400 MHz, D_2_O, 298.15K) of BtPy.


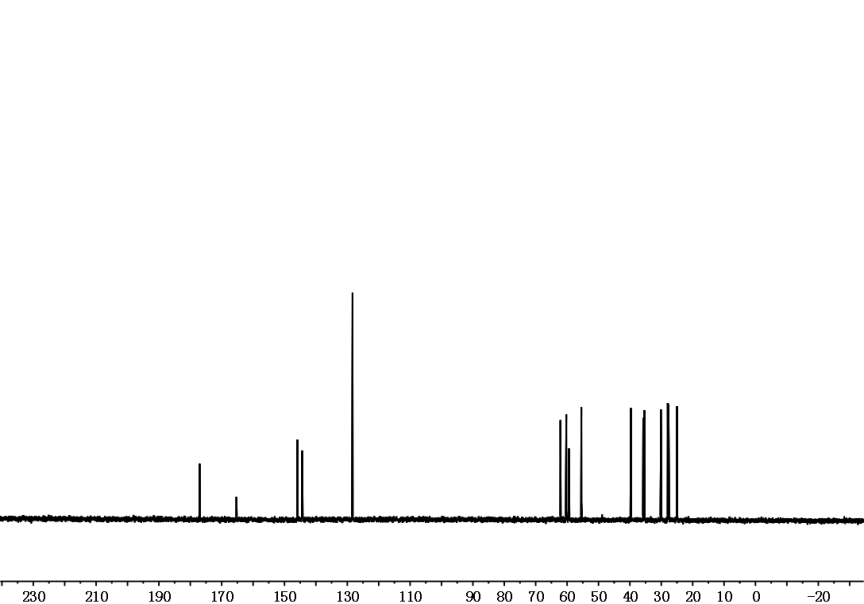


**Figure S2.** ^13^C NMR spectrum (400 MHz, D_2_O, 298.15K) of BtPy


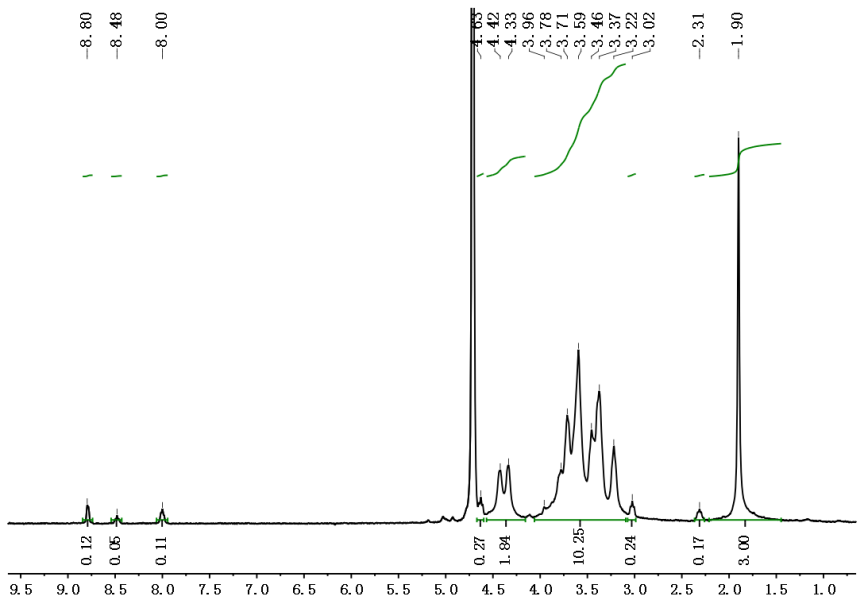


**Figure S3.** ^1^H NMR spectrum (400 MHz, D_2_O, 298.15K) of HAPy.


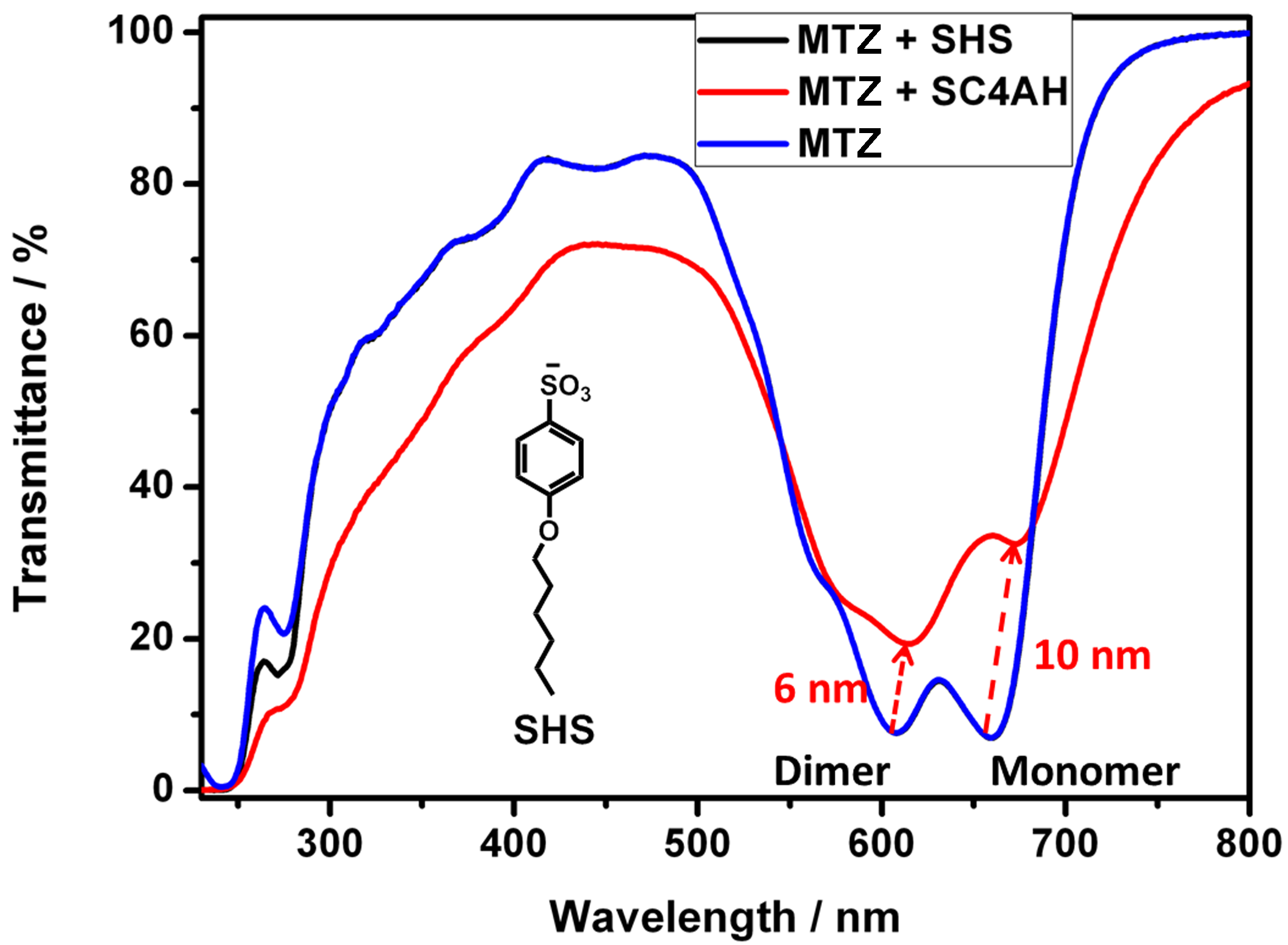
(a)
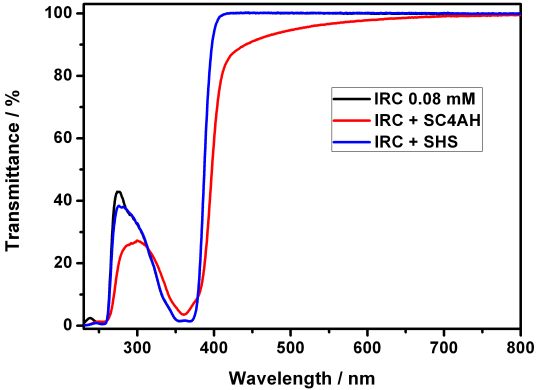
(b)

**Figure S4.** (a) Optical transmittance of free MTZ, SC4AH–MTZ, and SHS–MTZ. (b) Optical transmittance of free IRC, SC4AH–IRC, and SHS–IRC. [IRC] = 0.08 mM, [SC4AH] = 0.02 mM, [SHS] = 0.08 mM.


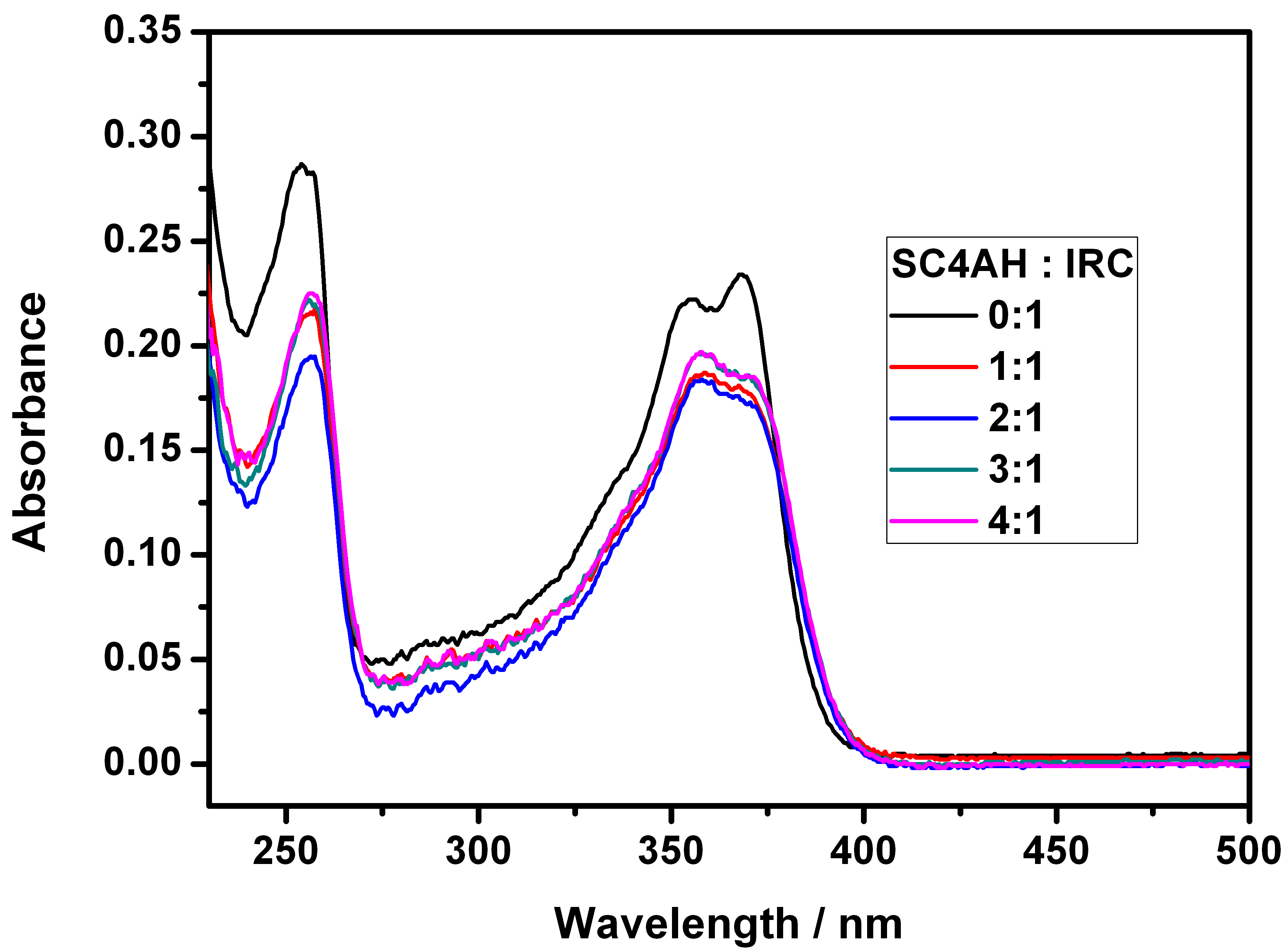
(a)
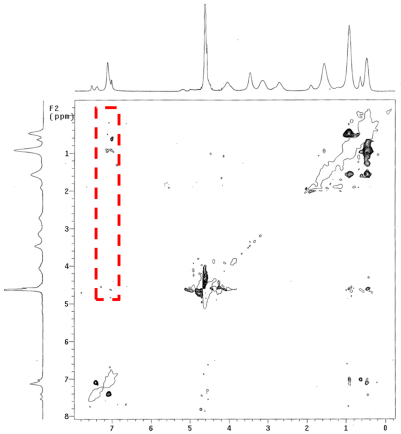
(b)

**Figure S5.** UV spectra of IRC in the absence and presence of SC4AH in solution. [IRC] = 0.01 mM. (b) 2D ROESY spectrum (300 MHz, D_2_O, 298.15K) of SC4AH–IRC complex. [SC4AH] = [IRC] = 5 mM.


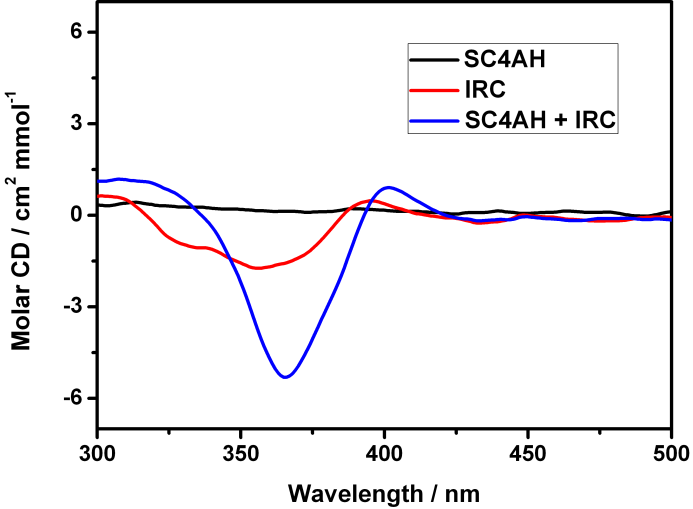


**Figure S6.** CD spectra of SC4AH, IRC and SC4AH–IRC coassembly. [IRC] = 0.04 mM, [SC4AH] = 0.01 mM.


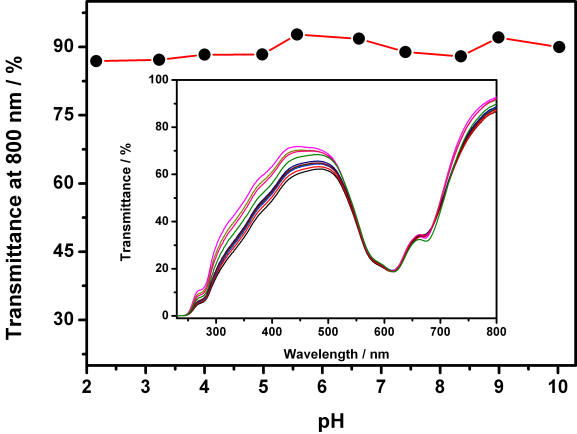
(a)
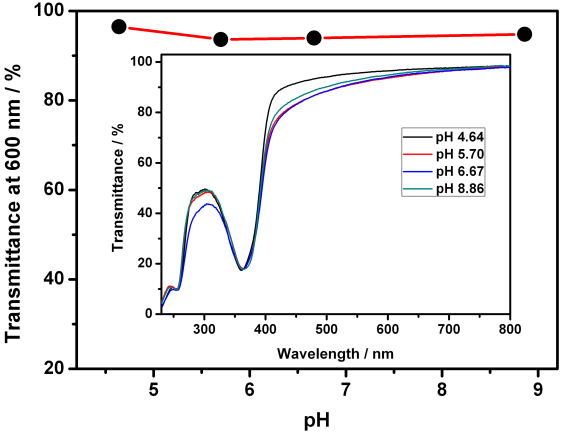
(b)

**Figure S7.** (a) Optical transmittance of SC4AH–MTZ coassembly at 800 nm at different pHs. (b) Optical transmittance of SC4AH–IRC coassembly at 600 nm at different pHs.


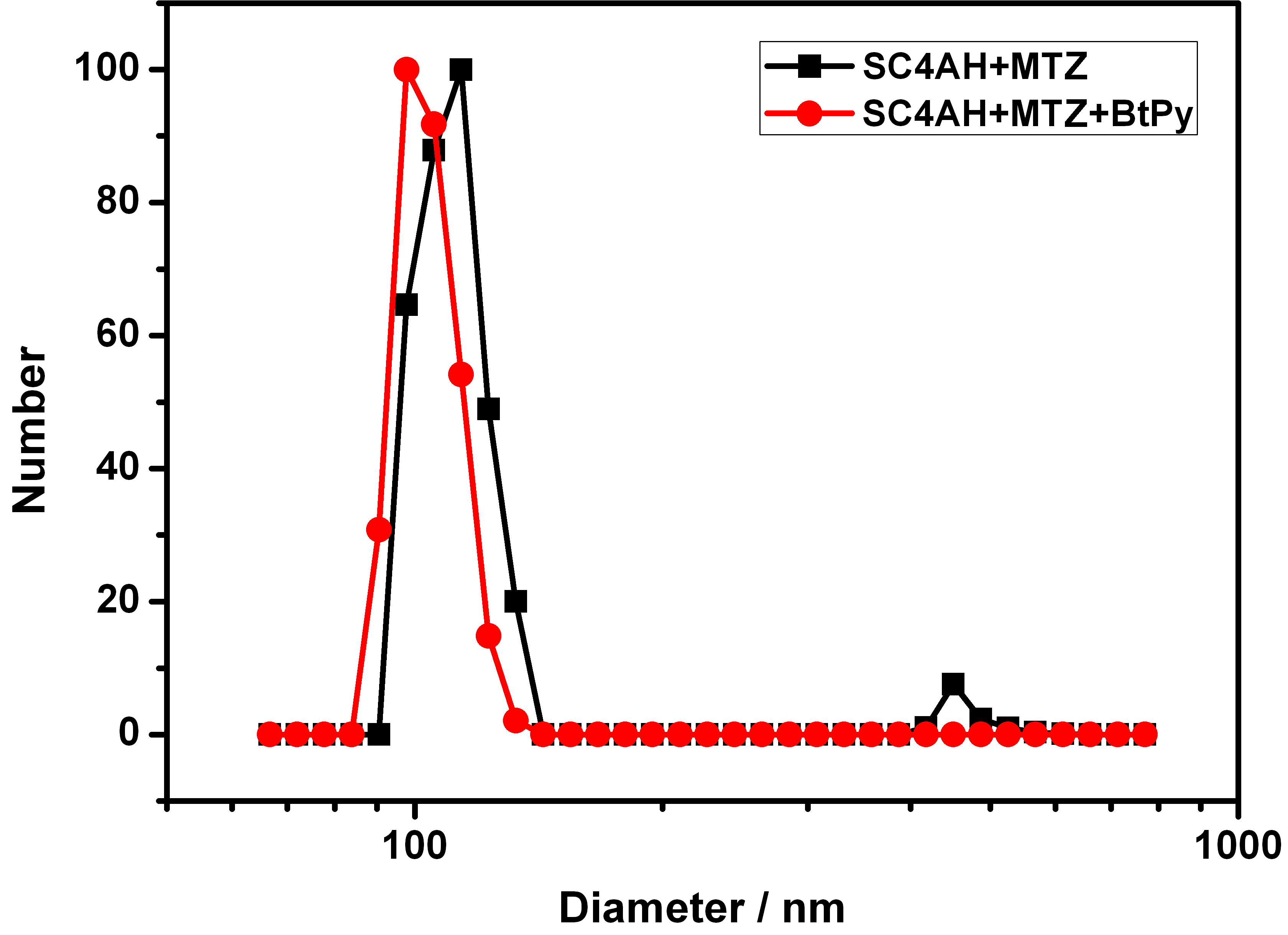
(a)
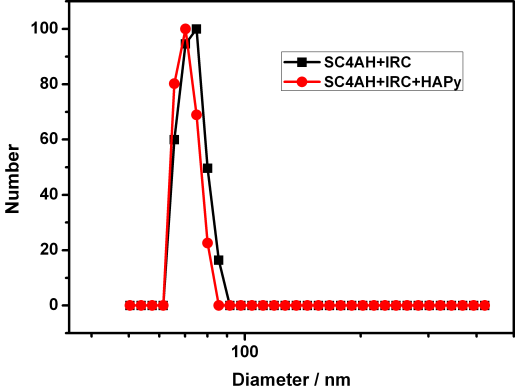
(b)

**Figure S8.** Size distributions determined by number of (a) SC4AH–MTZ coassembly with and without BtPy and (b) SC4AH–IRC coassembly with and without HAPy measured by DLS.


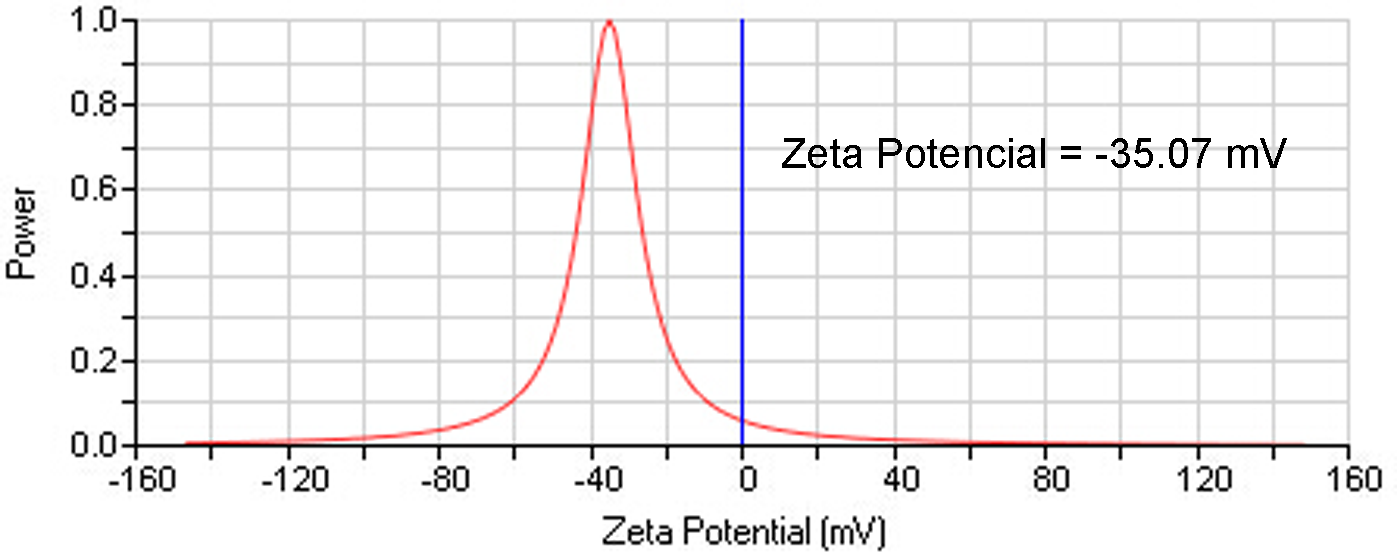
(a)
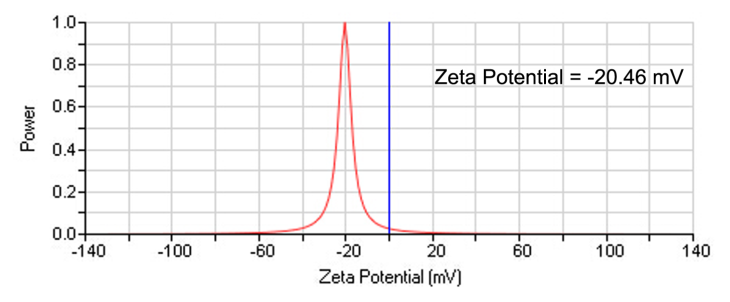
(b)

**Figure S9.** Zeta potential data of (a) SC4AH–MTZ coassembly and (b) SC4AH–IRC coassembly.

**^1^H NMR spectra of** **BtPy with SC4AH.** Since SC4AH–MTZ coassembly was precipitated at such a high concentration for NMR experiments, we only measured ^1^H NMR spectra of BtPy in the presence and absence of SC4AH to explore the binding behavior.


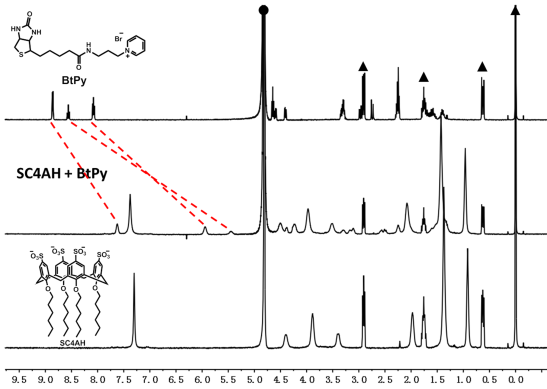
(a)
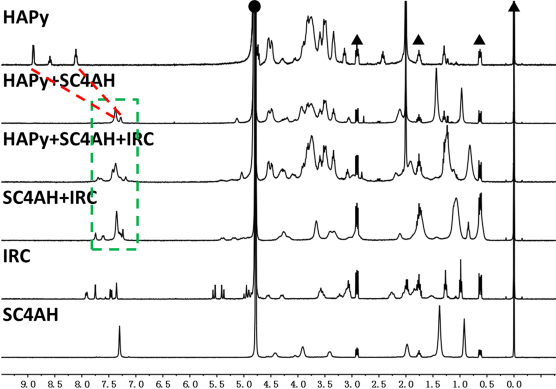
(b)

**Figure S10.** (a) ^1^H NMR spectra of BtPy in the presence and absence of SC4AH. [SC4AH] = [BtPy] = 1 mM. (b) ^1^H NMR spectra of HAPy, SC4AH–HAPy complex, SC4AH–HAPy–IRC complex, SC4AH–IRC complex, IRC and SC4AH in D_2_O. [SC4AH] = [IRC] = [HAPy] = 1 mM. DSS was added as an internal reference. The solvent (HDO) and DSS are denoted as symbols ● and ▲, respectively.


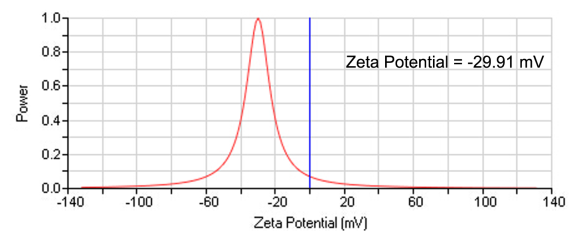
(a)
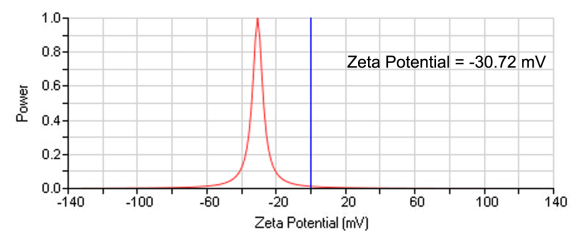
(b)

**Figure S11.** Zeta potential data of SC4AH–IRC coassembly in the presence of HAPy. (a) [HAPy] = 0.01 mM, (b) [HAPy] = 0.02 mM.


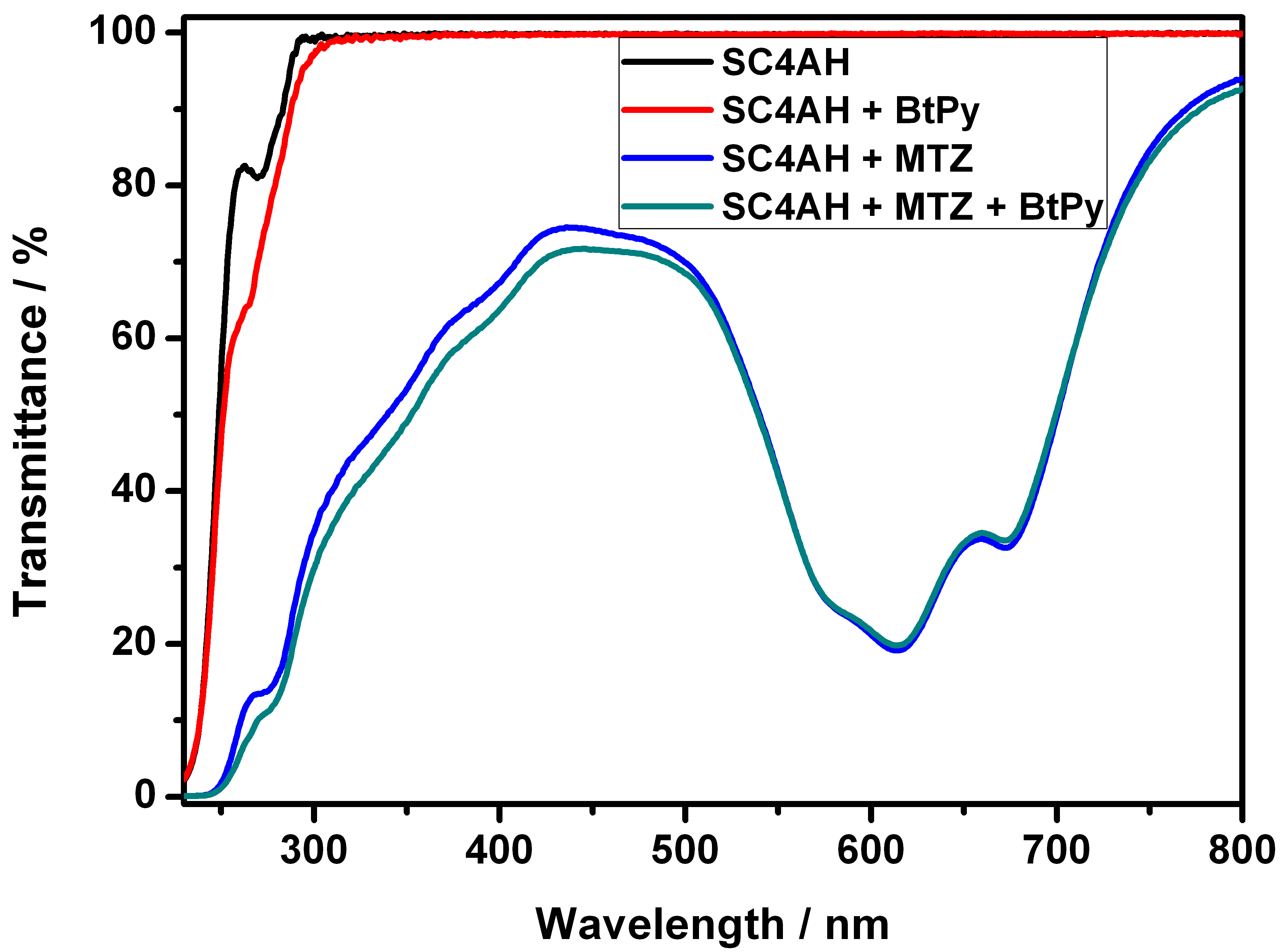
(a)
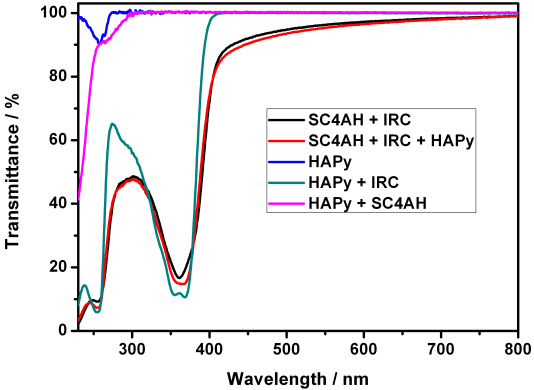
(b)

**Figure S12.** (a) Optical transmittance of free SC4AH, SC4AH–MTZ, SC4AH with BtPy and SC4AH–MTZ with BtPy. [SC4AH] = 0.04 mM, [MTZ] = 0.06 mM, [BtPy] = 0.04 mM. (b) Optical transmittance of SC4AH–IRC, SC4AH–IRC with HAPy, free HAPy, IRC with HAPy and SC4AH with HAPy. [IRC] = 0.04 mM, [SC4AH] = 0.01 mM, [HAPy] = 0.01 mM.


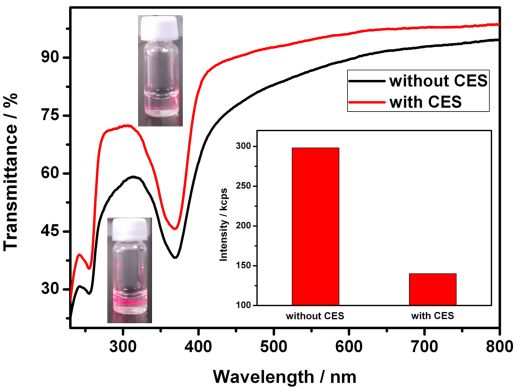
(a)
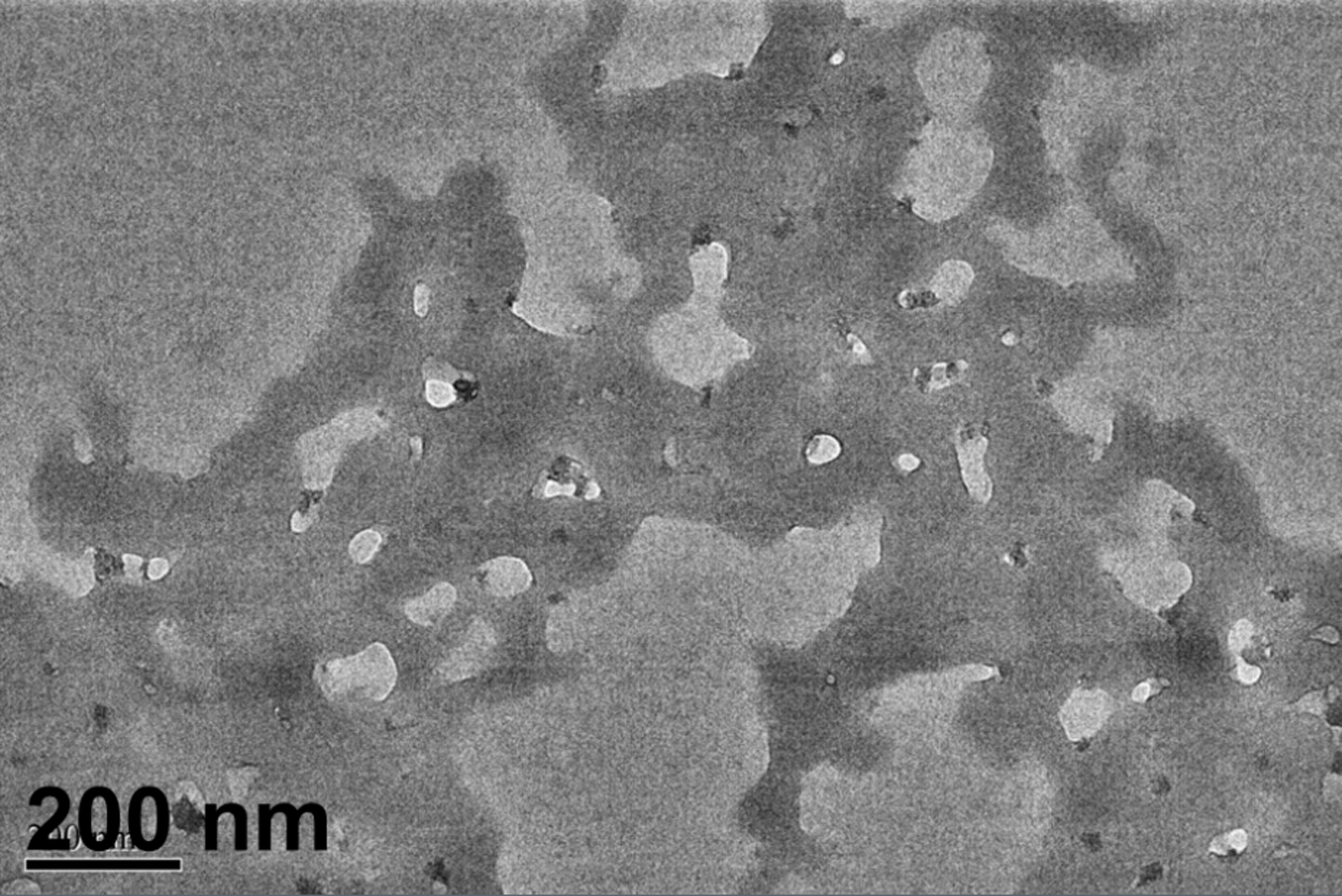
(b)

**Figure S13.** (a) Optical transmittance of SC4AH–IRC coassembly in the absence and presence of CES (0.33 ug / ml) at pH 7.6 for 20 h. Inset: Scattering-light intensity and tyndall effect of SC4AH–IRC coassembly with (down) and without (up) CES treatment. (b) High-resolution TEM image of SC4AH–IRC coassembly after hydrolyzation.


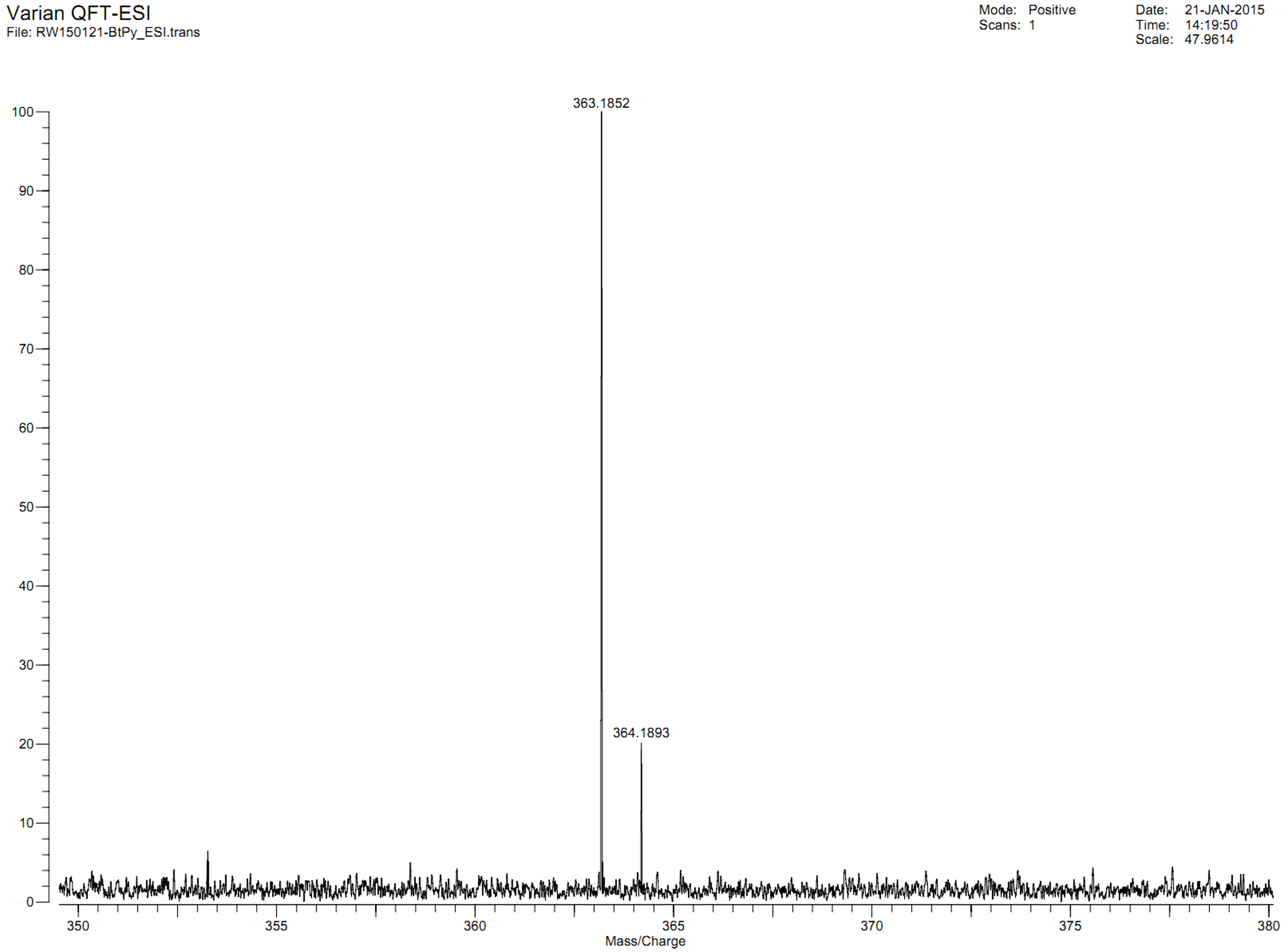


**Figure S14.** HR-MS spectrum of BtPy.

**Supplementary Information References**

1. . Qin, Z., Guo, D.-S., Gao, X.-N. & Liu, Y. Supra-amphiphilic aggregates formed by *p*-sulfonatocalix[4]arenes and the antipsychotic drug chlorpromazine. *Soft Matter* **10**, 2253−2263 (2014). [↑](#endnote-ref-2)
2. . Zhao, H.-X., Guo, D.-S. & Liu, Y. Binding Behaviors of *p*-Sulfonatocalix[4]arene with Gemini Guests. *J. Phys. Chem. B* **117**, 1978−1987 (2013). [↑](#endnote-ref-3)
3. . Yang, Y., Zhang, Y.-M., Chen, Y., Chen, J.-T. & Liu, Y. Targeted Polysaccharide Nanoparticle for Adamplatin Prodrug Delivery. *J. Med. Chem.* **56**, 9725−9736 (2013). [↑](#endnote-ref-4)
